# Psychometric properties of the Adolescent Motor Competence Questionnaire for Norwegian adolescents

**DOI:** 10.3389/fpsyg.2024.1296923

**Published:** 2024-01-23

**Authors:** Håvard Lorås, Monika Haga, Ruben Vist Hagen, Øyvind Bjerke, Amanda Timler, Ole Johan Sando

**Affiliations:** ^1^Department of Teacher Education, Faculty of Social and Educational Sciences, NTNU, Trondheim, Norway; ^2^School of Health Sciences and Physiotherapy, Faculty of Medicine, Nursing, Midwifery and Health Sciences, The University of Notre Dame, Fremantle, WA, Australia; ^3^Department of Physical Education and Health, Queen Maud University College of Early Childhood Education, Trondheim, Norway

**Keywords:** construct validity, internal consistency, measurement invariance, self-perception, motor skill

## Abstract

The objective of this study was to examine the psychometric properties of the Adolescent Motor Competence Questionnaire (AMCQ) for Norwegian adolescents. To this end, a sample of 349 Norwegian-speaking adolescents (13–16 years old) were recruited and completed the AMCQ. Initial results showed that confirmatory factor analysis (CFA) did not indicate statistical support for previous statistical models reported in the literature. Further analysis indicated factorial validity for a novel three-factor model identified through exploratory factor analysis, encompassing measures of fine motor skill (*α* = 0.65), gross motor skill (*α* = 0.74), and activities of daily living (ADL; *α* = 0.79) with acceptable internal consistency coefficients. Subsequent analysis indicated indices of measurement invariance in the study sample, as males rated their competence higher compared to females in 19 of the 27 items, and better model fit was obtained for the female adolescents. Strong invariance was tenable, and no factor mean differences were found across older and younger adolescents or across BMI scores. Overall results thus suggested that the AMCQ has acceptable psychometric properties and can be confidently used in further work with perceived motor competence in Norwegian 13–16 years-old adolescents.

## 1 Introduction

The concept of *perceived motor competence* refers to individual perceptions about the ability to execute movement skills, such as handwriting, kicking, and catching a ball (Missiuna, 1998; Raudsepp and Liblik, 2002). In general terms, it belongs to the multidimensional construct of self-perception, which is composed of a wide range of domains (e.g., social, emotional, cognitive, and academic), and more specifically, it belongs to the subdomain of perceived *physical* competence, which is broadly defined as perceptions of actual abilities (Harter, 1999). Perceived physical competence might also be considered as multidimensional, and perceived motor competence is thus a subdomain-specific self-perception of competence in motor behaviours executed in a variety of environments (Estevan and Barnett, 2018).

In theoretical models, perceived motor competence has been conceptualised as a mediator to explain how actual and perceived motor competence drives children and adolescents’ engagement in physical activities and team-based sports (Stodden et al., 2008; Robinson et al., 2015; Hulteen et al., 2018). Research from the past decade has indeed confirmed perceived motor competence as a mediator between actual motor competence and physical activity participation (De Meester et al., 2016; Jaakkola et al., 2019; Estevan et al., 2021). These findings suggest a synergistic nature of the relationship between actual and perceived motor competence that may promote either positive or negative trajectories of physical activity and health-related fitness resulting in healthy or unhealthy weight status (Stodden et al., 2008; Robinson et al., 2015).

Overall, the association between perceived motor competence and actual motor competence in adolescents is found to be generally small to moderate (De Meester et al., 2020; Philpott et al., 2020). As adolescent’s cognitive abilities become further enhanced and develop, they can potentially evaluate their own ability more accurately compared to younger children, especially by comparing themselves with their peers (Harter, 1999) and their parents (Timler et al., 2018a,b). Indeed, age is often hypothesised to moderate the relationship between motor competence and perceived motor competence/physical self-perception (Stodden et al., 2014), albeit an absence of a significant age effect may not necessarily indicate that adolescents are more precise in assessing their motor competence more accurately compared to younger children (De Meester et al., 2016, 2020). Furthermore, no gender differences in perceived motor competence are displayed in preschool children, but from the elementary school years onwards, boys typically report higher perceived motor competence than girls in which might reflect differences in participation rates in various types of physical activities and sport (Pesce et al., 2018).

Although both actual and perceived motor competence seem to play a crucial role in the development of a healthy and active lifestyle among individuals (Babic et al., 2014; Timo et al., 2016; Barnett et al., 2022), it is still unclear how these mechanisms become manifest in different subgroups (e.g., age). In particular, there is limited knowledge about perceived motor competence in adolescents *per se* (Lander et al., 2019; De Meester et al., 2020), and how it potentially affects physical activity engagement, and motivation for, or enjoyment of, sport and physical activities (Dreiskämper et al., 2022; Menescardi et al., 2022, 2023).

The connection between one’s perceived motor competence and engagement in physical activity appears to be influenced by intrinsic motivation, as indicated by the findings of Menescardi et al. (2022). This suggests that individuals who feel more competent are likely to enjoy and participate in physical activities to a greater extent. In addition, overestimation of competence in adolescents (i.e., high perceived motor competence) is associated with higher positive physical activity-related and motivational outcomes (De Meester et al., 2016). Individuals who overestimate their competence were also found to be significantly more autonomously motivated than their peers with similar levels of actual motor competence who accurately perceive their competence, which suggests that overestimation is a favourable phenomenon (De Meester et al., 2016). Perceived motor competence rather than actual motor competence also plays an essential role in early adolescents’ motivation for physical education (De Meester et al., 2016; Estevan et al., 2021).

Both actual and perceived motor competence could also potentially influence identity development in young people, with relationships found between the adolescent’s perceived level of motor competence and the health of their identity, as those with low motor competence form less healthy identities (Timler et al., 2020). Furthermore, self-perception in several domains (social competence, physical appearance, romantic appeal, behavioural conduct, close friendships, and global self-worth) mediated the negative impact of low perceived motor competence on identity health (Timler et al., 2019).

The *Adolescent Motor Competence Questionnaire* (AMCQ) is a self-report questionnaire developed by Timler et al. (2016) to identify the level of perceived motor competence and suspected motor difficulties among 12–18 years-old adolescents. It consists of 26 questions, scored on a 4-point Likert scale (never, sometimes, frequently, and always), with items reflecting a range of skills involving aspects of sports, schooling, self-care, activities of daily living, and fine/gross motor tasks typical of adolescents. The development of the AMCQ was informed by criteria A (coordinated motor skills is substantially below that expected given the chronological age) and B (motor skills deficits interferes with activities of daily living) for dyspraxia/development coordination disorder in the Diagnostic and Statistical Manual of Mental Disorders, Fifth Edition (DSM-5), as well as comments from samples of adolescents, key informants, and international experts (Timler et al., 2016).

The AMCQ was initially designed, developed, and further examined for aspects of psychometric validity with various samples of Australian adolescents. Concerning factorial validity, principal component analysis (PCA) on the 26 items indicated four factors that explain above half of the variance, conceptualised as *Participation in Physical Activity and Sports*, *Activities of Daily Living (ADL)*, *Public Performance,* and *Peer Comparison* (Timler et al., 2018a,b). In a different sample of Australian adolescents, however, a PCA extracted five factors that explained just below half of the statistical variance, representing *Ball Skills and Kinesthesis*, *ADL*, *Fine Motor and Gross Motor skills*, *Proprioception and Exteroception* and *Public Performance* (Plumb et al., 2021). There has not been reports of internal consistency reliability for these constructs through statistical metrics such as the Cronbach’s alpha (*α*), although high internal consistency (*α* = 0.9) has been reported for a summarized score across all items (Timler et al., 2016). The Total AMCQ score has been shown to be moderately correlated (*r* = 0.49) with actual motor competence [McCarron Assessment of Neuromuscular Development (MAND)], as well as with self-perceptions of constructs such as athletic competence, social competence, and physical appearance. Furthermore, the overall score has been shown to aid in the identification of suspected motor difficulties. These reports provide some evidence for convergent (i.e., associated with similar constructs) and divergent (i.e., not associated with different constructs of self-perception) validity (Timler et al., 2016, 2019, 2020).

The AMCQ psychometrics have, to a lesser extent, been examined across subpopulations of adolescents with different individual characteristics to investigate whether they can report systematic variations in perceptions of motor competence. Such systematic variations in perceptions can impact upon the content validity, and establishing the basic features of such measurement invariance is highly important, as strong invariance is an important pre-requisite for the application of mean subgroup comparisons, whereas strict invariance is a prerequisite for any form of group comparisons that rely on manifest scale scores rather than latent factors (Meredith, 1993; Millsap, 2011). Indeed, male adolescents have been found to report significantly higher self-perceptions on several of the AMCQ items, as well as on the total score, compared to female adolescents (Timler et al., 2018a,b). Plumb et al. (2021), however, did not find any significant difference between male and female adolescents on the AMCQ total score. To date, however, there is limited evidence that AMCQ can provide reliable information when used to compare subpopulations of adolescents presenting different attributes or characteristics. Additionally, none of the reviewed studies have examined any form of measurement invariance, or the presence of differential item functioning (i.e., the presence of systematic response tendencies that differ across groups) or possible latent mean differences in the AMCQ. It is therefore currently unknown whether the questionnaire could be used for any type of subgroup comparison as a function of adolescent’s age, body-mass index (BMI) or sex. As mentioned above, age and sex has been found to have an impact upon children and adolescent’s perceptions of their motor competence (Pesce et al., 2018; De Meester et al., 2020), and BMI is also included as a component in theoretical models targeting the relationship between actual and perceived motor competence and its role in developmental pathways towards health-related fitness (Stodden et al., 2008; Robinson et al., 2015). All these subgroup characteristics are thus considered important in research on perceived motor competence (De Meester et al., 2016).

### 1.1 Aim of the current study

Although actual motor competence is found to differ significantly across geographical regions (Bardid et al., 2015; Haga et al., 2018; Laukkanen et al., 2020), it is less clear about whether self-perception of motor competence in young people varies depending on culture and country of origin (Barnett and Goodway, 2018; Feitoza et al., 2018). So far, the Scandinavian countries in northern Europe have not seen a similar degree of validation efforts of perceived motor competence instruments available for adolescents, as compared to English-speaking countries. In Norway, the main language is one of the North Germanic languages (e.g., Norwegian, Swedish, Danish, and Icelandic), and a translation to this language thus represents a non-English validation. Norwegian adolescents’ experiences through participation in physical education, sport and/or physical activities are also quite different, compared to in other countries, and this might impact upon perceived motor competence. For example, a major part of Norwegian culture is connected to formal/informal outdoor activities across all seasons (e.g., swimming, cycling, and skiing), and, in terms of club memberships, the major organised sports for adolescents are cross-country skiing, soccer, and handball (NIH, 2021). However, Norway is experiencing a similar obesity epidemic and a decline in physical activity levels as seen across the rest of the world (WHO, 2022), which emphasises the importance of translating and validating instruments that measure perceived motor competence (such as the AMCQ) in order to gain a broader understanding of how this construct is associated with health behaviours (e.g., physical activity), and cognitive and affective factors, across geographic areas and cultures. The main objective of this study was therefore to examine the psychometric properties of the Norwegian adaptation of the AMCQ among a sample of Norwegian adolescents. To this end, the AMCQ was first translated, and then back translated and adapted to Norwegian. The factor validity and composite reliability of the proposed four and five-factor models were then examined (Timler et al., 2018a,b; Plumb et al., 2021), and alternative model fits were also explored. After establishing a model with an acceptable model fit, measurement invariance and the possible presence of systematic responses were examined as a function of an adolescent’s age, body-mass index (BMI) and sex.

## 2 Materials and methods

### 2.1 Participants

A convenience sample of 349 Norwegian-speaking adolescents was recruited from two secondary schools. The total sample size was considered appropriate for testing the proposed models of the AMCQ (Timler et al., 2018a,b; Plumb et al., 2021) with moderately sized factor loadings (~0.5) and examining the potential measurement invariance of the factor structure as a function of children’s sex, age, and BMI (Wolf et al., 2013). One of the schools was in an urban environment in a large Norwegian city, and one school was located in a rural setting. Before participating in the study, the adolescents and their teachers received information including statements about the purpose of the research, that participation was voluntary, and that all data were collected anonymously. As the study involved human participants, it was reviewed and approved by The Norwegian Social Science Data Services (project ID: 169464). The study was conducted in accordance with the local legislation and institutional requirements. Written informed consent to participate in this study was provided by the participants and participants legal guardian.

### 2.2 Procedure

#### 2.2.1 Translation and back-translation

The original AMCQ was translated and back-translated following a four-step process. First, the questionnaire was translated to a first draft in Norwegian by a motor behaviour expert (HL). This draft was back-translated by a native English-speaker, through a professional translating service. In the third step, another motor behaviour expert (MH) translated the English version provided by the translating service back to Norwegian. In the fourth step, the Norwegian back-translated version was discussed among the research group, and all translations/back-translations were sent to the scale’s developer (AT) for a final overview. Although most of the AMCQ items were readily translated to Norwegian language, a few items where culturally adapted to fit, and be understandable by, Norwegian adolescents (see Table 1).

**Table 1:** Overview of AMCQ items culturally adapted to Norwegian adolescents.

Original AMCQ item	Translation and adaptation	Comment
I prefer to participate in individual sports (such as swimming, martial arts, athletics) rather than team sports (such as football and netball)	I prefer to participate in individual sports (such as swimming, cross-country skiing, athletics) rather than team sports (such as football, handball, volleyball)	The examples of sports have been adjusted to reflect the most common individual and team sports among Norwegian adolescents
I prefer not to participate in sports at school	I prefer not to participate in physical education/sports at school	In Norwegian secondary schools, sports are primarily practiced in mandatory physical education classes. Both terms are therefore included
People say I am clumsy	Andre sier at jeg er klossete, klønete eller klumsete (In Norwegian)	As the word “clumsy” has no simple translation to Norwegian, three Norwegian terms were applied to clarify the meaning of the item
I can ride a bicycle	I can cycle fast on different surfaces and roads (such as asphalt, gravel, tractor road or forest path)	In Norway, the great majority learn to ride a bike at an early age. The item has therefore been culturally adapted to reflect variation in skill level among adolescents
I ask my friends and family for help to complete activities that require co-ordination (such as using a can opener)	I often ask friends or family to help me with things that require co-ordination (such as opening a jam jar, cutting bread etc.)	The example activity has been changed. A can opener is no longer among the most common tools in Norwegian households

#### 2.2.2 Adolescent Motor Competence Questionnaire

The AMCQ is a self-report motor competence questionnaire that was developed for adolescents between the ages of 12 and 18 years (Timler et al., 2016). It consists of 26 items examining the ecological presence of motor tasks and functional activities of daily living and was informed by the DSM-5 criteria A and B for Developmental Coordination Disorder (DCD; American Psychiatric Association, 2013). Participants respond on a 4-point Likert scale of Never (1), Sometimes (2), Frequently (3), and Always (4). To account for response bias, fifteen items are negatively worded. These are reverse scored for the analysis to Never (4), Sometimes (3), Frequently (2) and Always (1). A total score of 104 can be summed with a higher score indicating higher motor competence. The adolescents also provided self-report of their height (m), weight (kg), and sex.

#### 2.2.3 Protocol

Data were collected at participants’ schools during a 2 months period, and the AMCQ was completed in the classrooms with the pupils seated at their desk with a personal laptop. The questionnaire was presented through an online service (Nettskjema) that provides full anonymisation and concealment of information about the IP-addresses. Trained research assistants were present when the adolescents completed the AMCQ, to answer questions and resolve any technical difficulties with the online form, and they encouraged participants to answer as truthfully as possible.

### 2.3 Statistical analyses

Descriptive analysis was conducted to report the percentage of male and female adolescents’ ratings for each of the skills (i.e., “Never,” “Sometimes,” etc.). To investigate the relationship between self-ratings on each item and the adolescent’s age, sex, and BMI, regression analysis (Mehmetoglu and Jakobsen, 2017) was performed. Each item was regressed on the adolescent’s age, sex, and BMI. BMI was calculated by means of the conventional body weight/height^2^ formulae (Keys et al., 1972).

To evaluate the internal consistency and factor structure of the two previous proposed theoretical models by Plumb et al. (2021) and Timler et al. (2016), Cronbach’s alpha and confirmatory factor analysis (CFA) (Brown, 2014) was used. The evaluation of the models involves the utilisation of several indices, including the root mean square error of approximation (RMSEA), standardised root mean square residuals (SRMR), comparative fit index (CFI), and Tucker–Lewis index (TLI). We evaluated the proposed models based on the following criteria: RMSEA <0.08, SRMR <0.08, CFI >0.9 and TLI >0.9 (Hu and Bentler, 1999; Mehmetoglu and Jakobsen, 2017). To examine the factor loadings, we considered *R*^2^ estimates (item >0.25) and standardised factor loadings (item >0.40) (Brown, 2014). If the proposed theoretical model was found to not meet the specified acceptable model fit indices, alternative models were explored using exploratory factor analysis (EFA) (Brown, 2014).

The influence of sex, age, and BMI on the factor structures within the models was examined by multiple-groups CFA invariance evaluation (Brown, 2014) to determine whether the factor structure remained consistent across different groups (van de Schoot et al., 2015). For this analysis, the sample was divided into boys/girls, older/younger (median split at 15 years), and higher/lower BMI (median split at 20.5). This analysis enabled the assessment of whether the relationships between the observed indicators and the latent factors are comparable and invariant across various groups. In this study, the following analysis of measurement invariance was performed: (1) equal form, (2) equal factor loadings, (3) equal intercepts, (4) equal indicator residual variances, and (5) equal factor means (Brown, 2014). STATA MP version 18 (STATACorp) was used for all statistical analyses.

## 3 Results

The participating adolescents were between 13 and 16 years of age, and the average age was 14.6 years (SD = 0.6). The average BMI was 20.7 (SD = 2.8), ranging from 14.2 to 31.1. Among the participants, 169 identified as males, 174 as females and 6 as other genders. The six adolescents placed in the other category were not included in the analysis where gender was included. Descriptive statistics for the subgroups used for invariance testing revealed comparable BMIs between boys (20.6) and girls (20.7). The older participants had a slightly higher average BMI (21.1) than the younger participants (20.2). The youngest age group comprised 75 girls and 60 boys, while the oldest group comprised 99 girls and 100 boys. The most noteworthy demographic difference observed between the groups was related to the BMI of older versus younger participants.

The sum score of the 26 items indicated that the 349 pupils, on average, rated their motor competence as mostly good (*M* = 80, SD = 9.5), ranging between 50 and 100. Regression analysis with the sum score as the dependent variable demonstrates that boys have a higher sum score than girls (*B* = 6.2, *p* < 0.001). There was no statistically significant association between age or BMI and the sum score on the scale. Table 2 presents descriptive statistics and the percentage of boys’ and girls’ ratings for each item.

**Table 2:** Mean, standard deviation (SD) and percentage of male and female adolescents rating on each of the 26 items on the AMCQ (*n* = 349).

Items	Mean	SD	Never (%)		Sometimes (%)		Frequently (%)		Always (%)	
			Male	Female	Male	Female	Male	Female	Male	Female
Kick ball	2.6	0.9	5	10	33	51	37	28	25	11
Hit ball bat	2.6	0.8	4	8	28	52	51	34	17	6
Finish last^a^	2.6	1.1	13	33	18	28	28	28	41	11
Not clumsy	2.7	0.9	17	22	40	54	23	18	20	6
Individual sports	2.7	1.0	11	16	23	22	38	39	28	23
Throw ball	2.8	0.8	2	6	18	41	60	43	20	10
Handwriting readable	2.8	0.9	15	4	35	17	38	39	12	41
Handwriting fast	2.9	1.0	7	9	38	16	31	35	24	40
Coordinated like friends^a^	2.9	0.9	6	13	9	14	59	49	26	24
People say clumsy^a^	3.0	0.9	4	11	11	16	50	52	35	21
Bicycle	3.1	0.9	2	6	12	30	31	43	55	21
Bystander sport^a^	3.1	0.8	5	7	8	18	45	50	42	25
Scissors	3.1	0.9	5	5	23	17	37	31	35	48
Easy to get ready	3.2	0.9	2	6	14	32	25	24	59	38
Stumble stairs^a^	3.2	0.7	2	4	2	13	44	59	52	24
Break things^a^	3.2	0.9	5	7	7	12	38	41	50	40
Walk straight line^a^	3.2	0.8	4	5	11	9	43	47	42	39
Change clothes^a^	3.3	0.7	2	4	5	8	31	57	62	31
Learn new skills^a^	3.3	0.7	2	4	4	12	44	55	50	29
Flossing teeth	3.3	1.0	10	8	14	13	20	23	56	56
Help from friends^a^	3.4	0.8	3	3	6	15	23	43	68	39
Participate sport school^a^	3.4	1.0	8	8	7	11	9	32	76	49
Right and left^a^	3.5	0.8	2	6	2	12	15	31	81	51
Catch ball	3.5	0.7	1	1	8	15	23	37	68	47
Knife and fork^a^	3.7	0.6	2	2	2	3	11	16	85	79
Balance one leg	3.8	0.6	1	2	1	7	8	15	90	76

a Negatively formulated items that have been reversed.

Sex was significantly unrelated to three items: *using a knife and fork*, *walking in a straight line* and *flossing teeth*. Girls perceived their skills higher than boys on the *scissor* item (*B* = −0.21, *p* = 0.028), *readable handwriting* (*B* = −0.68, *p* < 0.001) and *fast handwriting* (*B* = −0.33, *p* < 0.001). However, boys scored significantly higher on the items *throwing a ball* (*B* = 0.40, *p* < 0.001), *participating in physical education at school* (*B* = 0.30, *p* = 0.003), *kicking a ball* (*B* = 0.43, *p* < 0.001), *being called clumsy* (*B* = 0.32, *p* < 0.001), *riding a bicycle* (*B* = 0.58, *p* < 0.001), *easy to get ready* (*B* = 0.46, *p* < 0.001), *catching a ball* (*B* = 0.29, *p* < 0.001), *hitting a ball with a bat* (*B* = 0.42, *p* < 0.001), *bystander sport* (*B* = 0.32, *p* < 0.001), *coordinated like friends* (*B* = 0.20, *p* = 0.036), *right and left* (*B* = 0.48, *p* < 0.001), *finishing last* (*B* = 0.80, *p* < 0.001), *help from friends* (*B* = 0.38, *p* < 0.001), *stumbling on stairs* (*B* = 0.42, *p* < 0.001), *changing clothes* (*B* = 0.37, *p* < 0.001), *breaking things* (*B* = 0.19, *p* = 0.045), *learning new skills* (*B* = 0.32, *p* < 0.001), *being clumsy* (*B* = 0.35, *p* < 0.001) and *balance on one leg* (*B* = 0.21, *p* < 0.001). These findings suggest substantial variation between boys and girls in the present sample regarding how well they rate their skills.

Older pupils scored significantly higher on items *using scissors* (*B* = 0.19, *p* = 0.026) and *not being clumsy* (*B* = 0.18, *p* = 0.038), while the other items were unrelated to the pupil’s age. Similarly, BMI was mostly unrelated to the scoring on the items. Pupils with a lower BMI scored significantly higher on the items *riding a bicycle* (*B* = −0.04, *p* = 0.008), *right and left* (*B* = −0.03, *p* = 0.021) and *balancing on one leg* (*B* = −0.05, *p* < 0.001). Age and BMI did not therefore seem to influence the scoring to any notable extent.

### 3.1 Examining proposed factor structures

Next, the proposed factor structures by Timler et al. (2018a,b) and Plumb et al. (2021) were evaluated. Cronbach’s alpha was utilised to assess the internal consistency reliability of the scales. The results revealed that the four-factor structure suggested by Timler displayed varying levels of internal consistency: participation in physical activity and sport (*α* = 0.75) showed acceptable consistency, activities of daily living (*α* = 0.61) and public performance (*α* = 0.67) demonstrated questionable internal consistency, and peer comparison (*α* = 0.40) displayed weak consistency. CFA was conducted to evaluate the proposed models further. The model fit measures for the four-factor Timler model suggested inadequate fit in the present data [*x*^2^ = 836 (293), *p* < 0.001, RMSEA = 0.073, SRMR = 0.091, CFI = 0.71, TLI = 0.68].

Furthermore, several standardised factor loadings were below the threshold of 0.40: one item in the participation in physical activity and sport scale, four in the activities of daily living scale, and two in the peer comparison scale. Regarding *R*^2^ estimates, six of 10 items in the participation in physical activity and sport scale, and two of seven items in the activities of daily living scale, fell below the threshold of 0.25. The public performance scale had two of five items below this limit, while one item in the peer comparison scale exceeded the threshold of 0.25 for *R*^2^ estimates. These findings indicate inadequate model fit and limited internal consistency for the proposed four-factor model in the present data.

Similarly, the five-factor solution suggested by Plumb et al. (2021) also demonstrated varying internal consistency: Ball skills and kinesthesis (*α* = 0.72) showed acceptable internal consistency, activities of daily living (*α* = 0.69) showed questionable internal consistency, while weak internal consistency was found in the subscales for fine motor and gross motor (*α* = 0.49), proprioception and exteroception (*α* = 0.40) and public performance (*α* = 0.56). The model fit measures for the five-factor Plumb model also indicated inadequate fit in the present data [*x*^2^ = 879 (289), *p* < 0.001, RMSEA = 0.077, SRMR = 0.091, CFI = 0.69, TLI = 0.65]. Analysis of the standardised factor loadings revealed that two items in the ball skills and kinesthesis scale and two items in the activities of daily living scale fell below the threshold of 0.40. Only two items in the fine and gross motor scales and the proprioception and exteroception scale met this criterion. Looking at the *R*^2^ estimates, four items in the ball skills and kinesthesis scale and three items in the activities of daily living scale met the threshold of 0.25. Two items in the fine motor and gross motor scale met this criterion, while none of the items in the proprioception and exteroception scale did. These findings indicate inadequate model fit and limited internal consistency for the proposed Plumb five-factor model.

### 3.2 Exploratory factor analysis

After observing the problematic model fit for the previously suggested models, an exploratory factor analysis (EFA) was conducted in order to address the issue and explore an alternative measurement model for the items. Initially, a principal factor analysis revealed three factors with eigenvalues above 1.0. Additional analyses were conducted to confirm the three-factor solution’s appropriateness, including a screen plot examination, assessment of extracted variance, and parallel analysis. These supplementary analyses provided support for the selection of a three-factor solution. The three factors were interpreted as follows: factor one was related to gross motor skills, factor two was associated with activities of daily living, and factor three was indicative of fine motor skills. Rotated factor loadings (orthogonal varimax) are found in Table 3. To choose the items for further analysis, a relatively low threshold of 0.20 was set for rotated factor loadings. This threshold was intentionally selected to include various activities in the measurement model. Based on this criterion, three items were excluded from the further investigation: *participation in individual sports, participating in sport at school and balancing on one leg*. Furthermore, each item was considered for theoretical alignment and substantive importance for the overarching concept. The items *easy to get ready* and *not being clumsy* were deemed inconsistent with the theoretical construct of gross motor skills and were consequently removed from further analysis. Similarly, the item *finishing last* was excluded as it was perceived to be outside the intended construct of activities of daily living. Ultimately, the factor analysis process resulted in 20 items supporting a three-factor solution consisting of gross motor skills (6 items), activities of daily living (9 items), and fine motor skills (5 items). These factors were further evaluated in subsequent analyses for internal consistency, model fit and model invariance.

**Table 3:** Rotated factor loadings and uniqueness for selected factors of the 26-item AMCQ (*n* = 349).

Items	Gross motor skills	Activities of daily living	Fine motor skills	Uniqueness
Throw ball	**0.713**	0.021	0.041	0.445
Catch ball	**0.707**	0.075	0.100	0.423
Hit ball bat	**0.617**	0.155	0.080	0.505
Kick ball	**0.591**	0.043	−0.006	0.590
Bicycle	**0.361**	0.033	0.173	0.662
Bystander sport	**0.296**	0.126	−0.007	0.575
^a^Easy to get ready	**0.269**	0.162	0.072	0.674
^a^Not clumsy	**0.216**	0.086	0.112	0.800
Coordinated like friends	0.032	**0.712**	0.081	0.443
People say clumsy	0.141	**0.700**	0.002	0.486
Break things	0.050	**0.572**	0.100	0.533
Stumble stairs	0.039	**0.475**	0.015	0.528
Change clothes	0.056	**0.317**	−0.044	0.685
Learn new skills	0.188	**0.286**	0.167	0.500
Walk straight line	0.049	**0.282**	0.170	0.700
Help from friends	0.107	**0.277**	0.042	0.604
Right and left	0.163	**0.252**	0.053	0.618
^a^Finish last	0.144	**0.240**	−0.053	0.684
Handwriting readable	0.073	−0.002	**0.668**	0.535
Scissors	0.010	0.071	**0.626**	0.574
Handwriting fast	0.076	0.126	**0.567**	0.602
Flossing teeth	0.036	0.066	**0.387**	0.690
Knife and fork	−0.005	0.182	**0.201**	0.652
^a^Individual sports	0.106	0.027	−0.038	0.834
^a^Participate sport school	0.193	0.143	0.034	0.618
^a^Balance one leg	0.165	0.059	0.093	0.698

Factor loadings >0.20 is bolded.

a Excluded from the factor in further analysis based upon statistical and theoretical considerations.

Analysis of Cronbach’s alpha demonstrated mostly acceptable internal consistency with gross motor skills (*α* = 0.74) and activities of daily living (*α* = 0.79) being above the commonly used threshold of 0.70 and with fine motor skills (*α* = 0.65) falling below this threshold. Next, the proposed model was evaluated using CFA. The exogenous latent variables were allowed to be correlated, and gross motor skills were positively associated with both activities of daily living (*β* = 0.36, *p* < 0.001) and fine motor skills (*β* = 0.26, *p* < 0.001). Activities of daily living and fine motor skills were also significantly positively associated (*β* = 0.33, *p* < 0.001). Boys scored significantly higher on the latent variables gross motor skills (*β* = 0.24, *p* < 0.001) and activities of daily living (*β* = 0.39, *p* < 0.001), while girls scored significantly higher on fine motor skills (*β* = −0.35, *p* < 0.001). The age and BMI of the pupil were not significantly associated with any of the latent constructs.

### 3.3 Final model

The model fit measures for the alternative three-factor model indicate a reasonable fit [*x*^2^ = 425 (167), *p* < 0.001, RMSEA = 0.067, SRMR = 0.077, CFI = 0.84, TLI = 0.81]. Upon closer examination, one item in the fine motor skills scale (*using a knife and fork*) showed a standardised factor loading below the commonly applied threshold of 0.40 (Table 4). Additionally, seven items including *bystander sport*, *riding a bicycle*, *changing clothes*, *walking in a straight line*, *help from friends*, *right and left*, and *flossing teeth*, had *R*^2^ estimates below the threshold of 0.25 (Table 4). The chi-square test indicated a significant deviation between the observed and expected values based on the specified model (*χ*^2^ = 425, df = 167, *p* < 0.001). While the RMSEA and SRMR values fall within what is generally considered an acceptable range (below 0.08), it is noteworthy that the CFI and TLI values do not reach the commonly accepted threshold for good fit, which is often set at 0.90 or higher. Despite these limitations, the model was deemed satisfactory for invariance testing and further analysis.

**Table 4:** Standardised factor loadings (*λ*) and *R*^2^ estimates from the three-factor solution of the 20-item AMCQ using CFA (*n* = 349).

Items	Gross motor skills		Activities of daily living		Fine motor skills	
	*λ*	*R^2^*	*λ*	*R^2^*	*λ*	*R^2^*
Throw ball	0.71	0.51				
Catch ball	0.75	0.57				
Hit ball bat	0.65	0.42				
Kick ball	0.59	0.34				
Bicycle	0.41	0.17				
Bystander sport	0.41	0.17				
Coordinated like friends			0.66	0.43		
People say clumsy			0.59	0.35		
Break things			0.67	0.45		
Stumble stairs			0.62	0.38		
Change clothes			0.44	0.20		
Learn new skills			0.55	0.30		
Walk straight line			0.42	0.17		
Help from friends			0.49	0.24		
Right and left			0.43	0.18		
Handwriting readable					0.63	0.40
Scissors					0.66	0.44
Handwriting fast					0.63	0.39
Flossing teeth					0.45	0.20
Knife and fork					0.32	0.10

We conducted a multi-group CFA using the proposed three-factor theoretical model to examine measurement invariance for the factor structure across boys and girls. Initially, separate models were fitted for each group, as presented in Table 5. The fit indices for boys were *x*^2^ = 322 (167), *p* < 0.001, RMSEA = 0.074, SRMR = 0.092, CFI = 0.76, and TLI = 0.74, while for girls they were *x*^2^ = 312 (167), *p* < 0.001, RMSEA = 0.071, SRMR = 0.086, CFI = 0.81, and TLI = 0.71. Examining configural invariance revealed no significant differences in the factor structure between boys and girls. However, differences in fit indices were observed despite the similarity in form. These differences in fit indices suggest an unequal measurement model between boys and girls. Subsequent tests were conducted to further examine measurement invariance. The results revealed significant differences in factor loadings [*χ*^2^diff = 38.9 (17), *p* < 0.001]. Since metric invariance was not met, scalar invariance was not tested. These findings indicate that while the form of the three-factor model is equivalent between boys and girls, the measurement properties, specifically the factor loadings, significantly differ between the two groups.

**Table 5:** The goodness-of-fit indexes of previous models and full and sub samples for the 20-item AMCQ three-factor model.

Model	*N*	*χ*^2^ (df)	CFI	TLI	RMSEA	SRMR
Timler et al. (2018a,b)	349	836 (293)	0.71	0.68	0.073	0.091
Plumb et al. (2021)	349	860 (289)	0.69	0.65	0.076	0.090
Three-factor model	349	425 (167)	0.84	0.81	0.067	0.077
Three-factor model boys	169	322 (167)	0.76	0.74	0.074	0.092
Three-factor model girls	174	312 (167)	0.81	0.78	0.071	0.086
Three-factor model BMI <20.5	169	312 (167)	0.80	0.78	0.072	0.082
Three-factor model BMI >20.5	180	320 (167)	0.83	0.81	0.072	0.094
Three-factor model younger pupils	146	267 (167)	0.83	0.81	0.064	0.087
Three-factor model older pupils	203	365 (167)	0.81	0.78	0.077	0.086

*χ*^2^, chi-square; df, degrees of freedom; CFI, comparative fit index; RMSEA, root mean square error of approximation; SRMR, standardised root mean residual; TLI, Tucker–Lewis index. Probability level *p* < 0.05.

After examining measurement invariance for BMI and age, the findings indicate a relatively similar model fit across different BMI groups and age groups. Examining configural invariance revealed no significant differences in the factor structure between BMI or age groups. The model for pupils with a BMI above 20.5 [*x*^2^ = 320 (167), *p* < 0.001, RMSEA = 0.072, SRMR = 0.094, CFI = 0.83, TLI = 0.81] demonstrated a relatively similar fit compared to the model for pupils with BMI below 20.5 [*x*^2^ = 312 (167), *p* < 0.001, RMSEA = 0.072, SRMR = 0.082, CFI = 0.80, TLI = 0.78]. The further analysis also demonstrated equal factor loadings [*χ*^2^diff = 25.4 (17), *p* = 0.09], intercepts [*χ*^2^diff = 20.1 (17), *p* = 0.27] and factor means [*χ*^2^diff = 0.6 (3), *p* = 0.90] between the two BMI groups. There was, however, a significant difference in error variances between the groups [*χ*^2^diff = 34.1 (20), *p* = 0.03]. Similar results were found for the effect of age on measurement invariance. The model fit indices for younger pupils [*x*^2^ = 267 (167), *p* < 0.001, RMSEA = 0.064, SRMR = 0.087, CFI = 0.83, TLI = 0.81] were similar to those for the older pupils [*x*^2^ = 365 (167), *p* < 0.001, RMSEA = 0.077, SRMR = 0.086, CFI = 0.81, TLI = 0.78]. The further analysis demonstrated equal factor loadings [*χ*^2^diff = 13.4 (17), *p* = 0.71], intercepts [*χ*^2^diff = 13.3 (17), *p* = 0.71] and factor means [*χ*^2^diff = 3.5 (3), *p* = 0.32] between the two age groups. A significant difference in error variances between the groups was found [*χ*^2^diff = 34.1 (20), *p* = 0.03]. Based on the results presented above, there is evidence to suggest a significant difference in error variances between the groups of pupils with different BMI and between the two age groups. Overall, however, the findings indicate that BMI and age do not substantially influence the factor structure.

## 4 Discussion

The principal aim of the current study was to verify psychometric aspects of the Norwegian adaptation of the AMCQ among a sample (*n* = 343) of 13–16 years-old adolescents from secondary schools. Our results indicated that the respondents in the sample rated their perceived motor competence primarily as good or very good [3 (frequently) or 4 (always) on the Likert scale], although a higher degree of inter-item variance (mean response <3) was observed in nine items (see Table 1). As depicted in Tables 2, 4, 5, our CFA indicated lack of statistical support for the previously observed models in two different Australian samples (Timler et al., 2018a,b; Plumb et al., 2021) among the current sample of Norwegian adolescents. EFA on all items instead suggested a three-factor model with internal consistency reliability coefficients in the range of 0.65–0.74 (Cronbach’s alpha), reflecting relatively moderate inter-item correlation coefficients within these subscales. The proposed factors encompassed measures of gross motor skills (6 items), activities of daily living (9 items), and fine motor skills (5 items). A CFA indicated moderate support and goodness-of-fit for this proposed three-factor model for the Norwegian version of the AMCQ (see Tables 4, 5). However, there were indices of measurement invariance in the study sample, as male adolescents rated their competence higher compared to females in 19 items, while females on the other hand, rated themselves higher compared to males in three fine motor skill items (using scissors, readable handwriting, and fast handwriting). The CFA indicated a slightly better model fit for female adolescents, although no differential measurement invariance or factor mean differences were found for BMI or age.

As indicated in Table 2, six of the items originally in the AMCQ were not included in the final proposed model (Table 4). Three of these items (*individual sports, participation in physical education in school,* and *balancing on one leg*) did not display a pattern of loadings on any of the factors in the model. Furthermore, several of these items also displayed considerable skewness towards adolescents reporting 3 or 4 on the Likert scale. For example, 98% of the male participants and 91% of the females reported high perceived competence on the *balancing on one leg item*, and such responses indicate an item-specific ceiling effect that contributes with very little variance towards the statical modelling. A similar effect might explain the lack of model fit for the *participation in physical education in school* item, as physical education is a mandatory school subject in the Norwegian curriculum and teachers are obligated to work to ensure the inclusion of everyone. Accordingly, previous investigations have reported that most Norwegian adolescents enjoy physical education in school (Moen et al., 2018). As for the item of *preferring individual sports*, this item might have an unclear association with perceived motor competence in Norwegian adolescents, provided that both individual and team sports with high demands for motor coordination and motor skill level are practiced by adolescents.

The results of the current study thus suggest that that six of the original AMCQ items could be further adapted for cultural and environmental factors. For instance, the items concerning participation in sport and physical education might be reworded to reflect whether the adolescents participate in leisure-time sport. In similarity with many other countries, a substantial number of Norwegian adolescents drop out of sport during secondary school (NIH, 2021), and there might be differences in perceptions of motor competence related to their participation patterns. In the context of physical education, being a mandatory school subject, one might provide examples of type of activities (e.g., swimming and/or skiing) that adolescents might perceive themselves as more or less competent in performing.

Three of the original AMCQ items were dismissed from further analysis on the grounds of unclear theoretical relevance for the suggested construct. The items *easy to get ready* and *not being clumsy* had moderate-to-low factor loadings on the suggested gross motor skill factor. These items were thus omitted from further analysis, as they are more conceptually aligned with components of ADL, compared to the gross motor tasks (i.e., throwing, catching, kicking, etc.). The opposite was true for the item *finish last*, which was omitted due to a small factor loading on the ADL factor, while it seems to be more conceptually convergent with the gross motor skill factor. It thus appears that these three items could be reworded to capture aspects of Norwegian adolescents perceived motor competence. The general term *clumsy* has no simple translation to Norwegian (see Table 1), so one might need to conceptualise the question towards specific tasks, allowing them to reflect upon their motor competence level in, e.g., various physical activities outside the realms of sport and physical education (e.g., parkour, skating, and/or dancing). Similarly, running races is rarely conducted by Norwegian adolescents unless they participate in track & field during their leisure time, thus other examples of activities might be implemented in rewording. Asking about whether they feel anxious about skiing downhill or ice skating for instance, might represent more culturally relevant activities in Norway. The final sixth item not included in the proposed 20-item model, *I find it easy to get ready to go out*, did not seem to capture variance that could be associated with the (expected) ADL factor. Albeit there is no culturally evident explanation for this finding, it might be that the item need rewording towards other ADL components more relevant for Norwegian adolescents, such as walking in various environments (e.g., crowded city vs. in nature) or transportation to school (walking, scootering, cycling).

As can be observed in Table 4, the CFA converged on three hypothesised factors termed *gross motor skills*, *ADL*, and *fine motor skills*. All demonstrated relatively good internal consistency reliability estimates in the range of 0.65–0.79. Acceptable range of model fit were found for some estimates (i.e., RMSEA/SRMR); however, some estimates did not reach the commonly accepted thresholds for goodness-of-fit (i.e., CFI/TLI). Perceived motor competence captured by the AMCQ is based upon self-evaluation of individual performance levels across many different motor tasks, and it is well-known that assessment of actual motor performance is particularly prone to substantial inter-and intra-individual variability that, among other things, can be displayed as low correlations between performance for different motor tasks (Lorås and Sigmundsson, 2012; Sigmundsson et al., 2021). Furthermore, within-trial individual variability can be more substantial for motor tasks compared with cognitive tasks (Lövdén et al., 2008), which suggests that internal consistency statistics both within and between performance domains are not necessarily comparable. In view of the diverse nature of the motor tasks by which adolescents self-evaluate their competence level in AMCQ, one cannot necessarily expect consistent ratings of perceived motor competence level across all items within a specific factor.

The perceptions of competence in gross motor skills were encompassed by items associated with motor tasks that are well-known in the motor development literature (e.g., throwing, catching, and kicking). For younger children, these motor tasks are typically found in the conceptualisation of gross motor competence, which is often specified as level of proficiency in a range of fundamental movement skills that involve large muscle movements (Estevan and Barnett, 2018). Furthermore, the AMCQ items in the current study associated with the gross motor skill factor are also commonly found in widely applied assessment batteries of actual motor competence validated for adolescents (Hulteen et al., 2020). Similarly, the items encompassing the fine motor skill factor (see Table 2) also adhere to the conceptualisation of such skills as relating to hand and finger dexterity involving coordination of smaller muscles. In particular, handwriting and using scissors are also skills that are typically found in motor competence assessment batteries (Matheis and Estabillo, 2018).

The third factor emerging from the CFA was loaded by nine items associated with ADL. ADL in adolescents refers to tasks that are fundamental to supporting participation across school, home, and community environments (James et al., 2014). ADL is conceptualised in the “Activities and Participation” domain of the International Classification of Functioning (ICF) from the World Health Organization (WHO) and is defined here as life-skills required for self-care and self-maintenance.^1^ In the ICF, these tasks are classified as either (1) personal ADL tasks, which are oriented towards self-care, or (2) instrumental ADL tasks, which are oriented towards sustaining independence and require a higher level of physical and cognitive competency than personal ADL. The nine items representing the ADL factor in the current analysis seem to relate to both classifications—e.g., the item “*changing clothes*” represents a personal ADL task, and the item “*learning new skills*” represents an instrumental ADL task—so the AMCQ therefore potentially provides a broad conceptual assessment of perceived motor competence in ADL contexts. The presence of an ADL factor is also aligned with previous validation efforts of the AMCQ in Australian adolescents, although with some minor differences in the number of items: in the Plumb et al. (2021) and Timler et al. (2018a,b) models, seven items were loaded on the ADL factor, and there was not 100% convergence in the two Australian samples as to what items were associated with the ADL dimension (Timler et al., 2018a,b; Plumb et al., 2021).

Our results are generally consistent with those from previous validation efforts concerning perceived motor competence in both children and adolescents, in that males typically perceive themselves as having more competence than females (De Meester et al., 2020). In the current sample of Norwegian adolescents, males also reported higher perceived motor competence in 19 items in the AMCQ, while the female adolescents reported higher perceived competence in fine motor skills. These differences in perceptions of competence align with differences in measures of actual motor competence: in a meta-analysis of scores from 25 different countries across six continents, weighted mean scores indicated that the males in each age range exhibited higher levels of motor competence compared to their female counterparts (Bolger et al., 2021). Thus, sex has been found to be a consistent correlate for certain aspects of motor competence (Barnett et al., 2016). The results might therefore reflect that, in the current sample of Norwegian children, the measurement properties of the three-factor model differed significantly as a function of sex: CFA suggested a better fit for the three-factor model in females (see Table 4). The reason for this lack of measurement invariance across sex awaits further study, it might be speculated that this relates to overall differences in in what male and female adolescents value as important for their self-perceptions (Beyer, 1990; Pajares and Valiante, 1999). Overall, this suggests that decisions concerning comparisons between female and male adolescents using latent AMCQ variables must be based upon sample-specific validation efforts, relating to whether any measurement non-invariance needs to be accounted for in further statistical procedures.

In the current study, no impact of age was found on measurement invariance (see Table 4). These results therefore indicate that observed and latent scores of the AMCQ can be confidently used to compare adolescents as a function of their age in the range of 13–16 years old. Furthermore, item-specific analysis indicated that scores were mostly unrelated to the age of adolescents. Similarly, there were no indications of BMI as a potential contributor to measurement invariance or being significantly associated with item-specific scores. These results should be evaluated against the relatively moderate age range in the current sample (13–15 years), as well as BMI scores being predominantly in the normal range (Swallen et al., 2005). Also, based upon theoretical perspectives on self-perception, younger children might display inflated and less consistent responses, as they have less experience and awareness of peer-to-peer comparisons and reflections upon their own motor competence level (Maïano et al., 2022), which is established predominantly in adolescents (Harter, 2003). Indeed, in a meta-analysis of 69 studies on the association between actual motor competence and perceived motor competence/physical self-perception, significant pooled effects were found for locomotor, object control, stability/balance, and sport-specific competence, in which age (3–24 years old) did not appear as a significant moderator for any of the domains (De Meester et al., 2020). The authors did not therefore find support for less consistent perceptions even among younger children.

The AMCQ was initially introduced as a self-reported measurement tool for the identification of individuals with suspected motor difficulties in adolescent populations, stressing the importance of adequate motor proficiency in relation to appropriate physical health and leading an active lifestyle (Timler et al., 2016; Plumb et al., 2021). Argumentation for its use was therefore originally given a clinical purpose, rather than assessing motor competence as a central factor of human life-span development (Lopes et al., 2021). However, such easy to administer, and potentially reliable and valid means of assessing adolescents’ self-perceptions of motor competence provide an opportunity to collect data in large samples. Furthermore, it allows for addressing the lack of longitudinal studies examining the dynamic and complex interaction of other developmental factors associated with motor competence during young individuals’ development between childhood and adolescence (Lopes et al., 2021). Additionally, motor learning is an important aim in most physical education curricula (Lorås, 2020), so the assessment of both associations and effects of curricula on self-perceptions of motor competence, and of how levels of motor competence influence academic achievement in physical education, are of interest in the educational context of adolescents.

The current study has methodological limitations that warrant further investigation. First, no evidence of the test-retest reliability and aspects of concurrent/divergent/predictive validity of the Norwegian version of the AMCQ was proposed in the present study. These psychometric properties are currently being investigated among Norwegian adolescents with combinations of other self-report measures. A strength of the current study is that it represents the first validated tool for Norwegian adolescents to self-report their perceived motor competence, and among the first validation efforts for the AMCQ in non-English speaking adolescents. The proposed 20-item model with three factors is also conceptually convergent with self-report instruments designed for children (Estevan and Barnett, 2018), allowing for similar constructs to be examined across childhood and adolescence.

## 5 Conclusion

In conclusion, the results from the current study indicated that the psychometric properties (factor validity, internal consistency reliability, and measurement invariance) of the Norwegian translation of the AMCQ are sufficient for further work with perceived motor competence and some of its underlying constructs (fine motor skills, gross motor skills, activities of daily living) in Norwegian 13–16-year-old adolescents. Furthermore, the present study suggests that gender potentially impacts measurement invariance and factorial differences when adolescents rate their perceived motor competence, and this therefore needs to be accounted for in studies of this construct.

## Data availability statement

The raw data supporting the conclusions of this article will be made available by the authors, without undue reservation.

## Ethics statement

The studies involving human participants were reviewed and approved by the Ethics Committee of The Norwegian Social Science Data Services. The studies were conducted in accordance with the local legislation and institutional requirements. The participants provided their written informed consent to participate in this study.

## Author contributions

HL: Conceptualization, Data curation, Formal analysis, Investigation, Methodology, Project administration, Supervision, Visualization, Writing – original draft, Writing – review & editing. MH: Conceptualization, Writing – original draft, Writing – review & editing. RH: Conceptualization, Investigation, Methodology, Project administration, Supervision, Writing – original draft, Writing – review & editing. ØB: Conceptualization, Writing – original draft, Writing – review & editing. AT: Conceptualization, Methodology, Writing – original draft, Writing – review & editing. OS: Data curation, Formal analysis, Methodology, Writing – original draft, Writing – review & editing.

## Footnotes

^1^icd.who.int./dev11/l-icf/en
